# Physiological reactivity at rest and in response to social or emotional stimuli after a traumatic brain injury: A systematic review

**DOI:** 10.3389/fpsyg.2023.930177

**Published:** 2023-02-10

**Authors:** Alice Bodart, Sandra Invernizzi, Laurent Lefebvre, Mandy Rossignol

**Affiliations:** Cognitive Psychology and Neuropsychology Laboratory, Department of Psychology and Educational Sciences, University of Mons, Mons, Belgium

**Keywords:** traumatic brain injury, brain injury, physiological reactivity, autonomic reactivity, emotion, social stimuli

## Abstract

Numerous studies have shown that alterations in physiological reactivity (PR) after traumatic brain injury (TBI) are possibly associated with emotional deficits. We conducted a systematic review of these studies that evaluated PR in adults with moderate-to-severe TBI, either at rest or in response to emotional, stressful, or social stimuli. We focused on the most common measures of physiological response, including heart rate (HR), heart rate variability (HRV), respiratory sinus arrhythmia (RSA), electrodermal activity (EDA), salivary cortisol, facial electromyography (EMG), and blink reflex.

**Methods:**

A systematic literature search was conducted across six databases (PsycINFO, Psycarticles, SciencDirect, Cochrane Library, PubMed, and Scopus). The search returned 286 articles and 18 studies met the inclusion criteria.

**Results:**

Discrepancies were observed according to the type of physiological measure. Reduced physiological responses in patients with TBI have been reported in most EDA studies, which were also overrepresented in the review. In terms of facial EMG, patients with TBI appear to exhibit reduced activity of the corrugator muscle and diminished blink reflex, while in most studies, zygomaticus contraction did not show significant differences between TBI and controls. Interestingly, most studies measuring cardiac activity did not find significant differences between TBI and controls. Finally, one study measured salivary cortisol levels and reported no difference between patients with TBI and controls.

**Conclusion:**

Although disturbed EDA responses were frequently reported in patients with TBI, other measures did not consistently indicate an impairment in PR. These discrepancies could be due to the lesion pattern resulting from TBI, which could affect the PR to aversive stimuli. In addition, methodological differences concerning the measurements and their standardization as well as the characteristics of the patients may also be involved in these discrepancies. We propose methodological recommendations for the use of multiple and simultaneous PR measurements and standardization. Future research should converge toward a common methodology in terms of physiological data analysis to improve inter-study comparisons.

## 1. Introduction

Moderate to severe traumatic brain injury (TBI) can cause focal injuries at the site of the impact or in tissues opposite to the impact. Moreover, rapid acceleration and deceleration of the brain within the skull produces diffuse brain injuries characterized by widely distributed damage to axons, diffuse vascular injury, hypoxic–ischemic injury, and brain swelling (oedema; Andriessen et al., 2010). These injuries have a propensity to cause damage to the ventral frontal and temporal cortices (Stuss, 2011). The major sequelae of the damage are persistent emotional and behavioral disorders present in 62% of patients one year after TBI (Deb et al., 1999; Stéfan and Mathé, 2016). This damage is, in part, responsible for persistent emotional and behavioral disorders that disturb daily functioning, socio-professional reintegration, and quality of life (Milders et al., 2003). Among these difficulties, emotion regulation disorders result in emotional lability, indifference, and irritability (McDonald, 2013). Patients also report a decrease in their ability to experience emotional states, such as sadness or fear (Croker and McDonald, 2005), and the degree of impairment in subjective emotional experience is correlated with the severity of social behavioral problems (Hornak et al., 1996). Compared with motor and cognitive sequelae, emotional disorders have a greater impact on social reintegration (Milders et al., 2003). As these disorders are among the most frequent sequelae, it is crucial to investigate their etiology and propose ways to remedy them.

Multilevel models of emotional response postulate that its adequate expression is based on the awareness of the emotion experienced, which in turn is based on the ability to become aware of the bodily changes associated with the emotion (Lane, 2000). These responses refer to physiological reactivity (PR). The implications of PR in emotions have been discussed for more than a century. The first major peripheralist theory of James and Lange (James, 1884) postulated that physiological changes [including facial expressions (crying, smiling, blinking) and peripheral visceral responses (heart rate, emotional sweating, etc.)] elicited by the stimulus are at the origin of the emotional subjective feeling. This theory was strongly criticized by Cannon (1931), who postulated independence between PR and emotion. Modern theories posit that the role of PR in emotional processes lies at the intersection of these two theories (Scherer, 2005). Indeed, PR should be considered a cue, among others, on which the formation of emotion is based (Christopoulos et al., 2019). Damasio’s somatic marker hypothesis places the perception of somatic states at the center of emotional reasoning and interpersonal relationships (Damasio et al., 1996). According to this hypothesis, the prefrontal cortex records somatic states experienced during each emotional experience in the form of internal representations called somatic markers. These markers are reactivated upon subsequent confrontations with similar situations/stimuli to adapt behavior for predictable consequences. This hypothesis is based on the famous case of Phineas Gage who, following a severe TBI with prefrontal lesions, developed emotional behavioral disorders similar to an ‘acquired sociopathy’. Therefore, this theory supports the involvement of PR in emotional processes.

**Figure 1:**
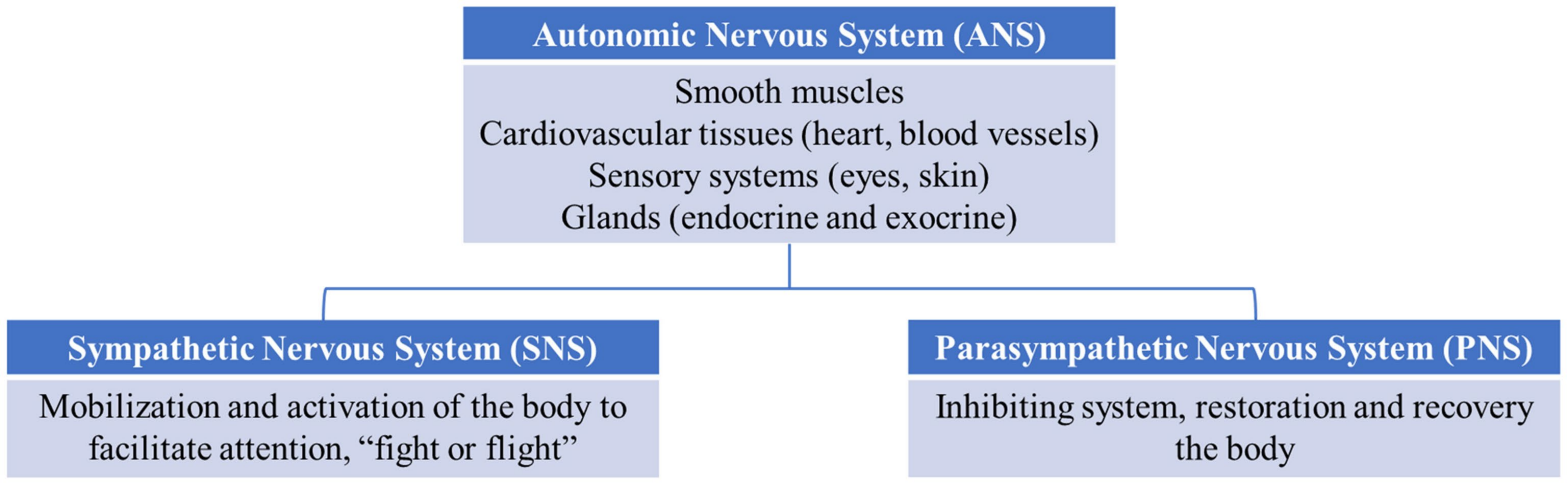
Autonomic nervous system divisions.

The PR reflects the electrical and hormonal expression of autonomic activity under the control of the autonomic nervous system (ANS) and limbic–hypothalamic–pituitary–adrenal axis (LHPA). The ANS is part of the nervous system which controls the automatic functions of the body, such as smooth muscles, cardiovascular tissues (heart, blood vessels), sensory systems (eyes, skin), and glands (endocrine and exocrine), to maintain internal homeostasis and adapt it to environmental changes (see Figure 1). The ANS comprises two branches: the sympathetic nervous system (SNS) and parasympathetic nervous system (PNS). The SNS is an activating system for mobilization and activation of the body to facilitate attention, fight, or flight. The PNS is an inhibiting system that allows the restoration and recovery of the body. The ANS activation in response to stimulation produces changes in the heart rate (HR), heart rate variability (HRV), and electrodermal activity (EDA), whereas limbic–hypothalamic–pituitary–adrenal axis (LHPA) activation produces the stress hormone cortisol. The ANS is controlled by a neuronal system composed of the hypothalamus, limbic system, and frontal lobe areas (Christopoulos et al., 2019). Accordingly, impairments in PR are frequently reported following TBI and are not surprising given the location of the lesions. Several studies have reported reduced startle blinks, skin conductance activity, and facial reactivity to emotional pictures and movies (Sánchez-Navarro et al., 2005; Soussignan et al., 2005; Saunders et al., 2006; de Sousa et al., 2010, 2011). Several researchers have hypothesized that abnormalities in PR may underlie the emotional issues in TBI (de Sousa et al., 2010; Rushby et al., 2013b; Francis et al., 2016). In addition to research on TBI populations, other research and reviews have reported links between PR abnormalities and different psychiatric disorders, such as anxiety (Hyde et al., 2019), depression (Sarchiapone et al., 2018), and behavioral disorders, such as aggression, psychopathy, and conduct problems (Lorber, 2004).

Given the role of PR in emotional processes, PR abnormalities may play a major role in the etiology of emotional difficulties after TBI. These difficulties manifest as emotional regulation disorders, such as emotional lability, indifference, or irritability (McDonald, 2013). Patients also report a decrease in their ability to experience emotional states such as sadness or fear (Croker and McDonald, 2005), with the degree of impairment in subjective emotional experience correlating with the severity of social behavioral problems (Hornak et al., 1996). Although these disorders are known to exist, to date, no systematic review of the literature on physiological reactivity abnormalities in TBI has been conducted. Therefore, this review will focus on potential physiological disturbances at rest or in response to emotional, social, and stressful stimuli in adults with moderate-to-severe TBI compared to healthy controls. Social and emotional stimuli refer to all stimuli and tasks involving other people, elements of social interactions (gaze or speech), or human emotions. Stressors refer to all situations that may induce stress in the participants. This review includes the most common measures of physiological responses, including HR, HRV, RSA, EDA, EMG, and blink reflex. For research involving resting physiological data collection, only studies linking physiological data to psychological variables measured using cognitive tasks, self-report, or hetero-report psychometric scales were selected.

## 2. Methods

### 2.1. Protocol and registration

Before starting the research procedures, the protocol for this review was submitted to the PROSPERO International Prospective Register of Systematic Reviews in July 2021 (registration number CRD42021266886). This protocol details the complete methodology of this review, and no changes were made during the review.

### 2.2. Search procedure

We conducted a systematic review of the literature in accordance with the PRISMA (Preferred Reporting Items for Systematic Reviews and Meta-Analyzes) guidelines. The research was conducted using PsycINFO, Psycarticles, SciencDirect, Cochrane Library, PubMed, and Scopus. The search was limited to peer-reviewed articles in French and English from 2000 to 2021. Keywords in the title or abstract were “*traumatic brain injury” OR “chronic brain injury” OR “brain injury” OR “head injury” OR “head trauma*” in combination with the following keywords: *“physiological” OR “physiological reactivity” OR “physiological change” OR “physiological response” OR “arousal” OR “skin conductance” OR “heart rate” OR “heart rate variability” OR “facial reactivity” OR “electrodermal” OR “galvanic skin response” OR “arousal” OR “hyperarousal” OR “hypoarousal” OR “autonomic” OR “eyeblink startle*” and “emotion” OR “stimuli” OR “emotional responses” OR “emotional reactivity” OR “rest” OR “stress” OR “habituation.”

#### 2.2.1. Inclusion and exclusion criterion

We included studies that (1) had at least one participant with moderate to severe TBI; (2) compared the physiological data of participants with TBI to a group of healthy participants without TBI or other psychiatric or neurologic histories; (3) measured at least one of HR, HRV, RSA, EDA, facial EMG, startle blink, or cortisol; (4) used research designs that exposed participant(s) to at least one stimulus condition different from baseline; or (5) involved resting physiological data collection, linking physiological data to psychological variables measured using cognitive tasks, self-report, or hetero-report psychometric scales. We excluded studies if they (1) are animal studies; (2) utilized physiological measures to assess the efficiency of a pharmacological intervention; (3) included participants under 18 or over 80; (4) included patients in a persistent vegetative state; (5) targeted pathologies other than moderate to severe TBI (post-traumatic stress disorder, mild TBI,TBI without brain lesion or cognitive and emotional sequelae, other neurological, post-traumatic stress disorder); (6) had no control group; (7) involved no physiological measures; and (8) are conference papers because they are not always peer-reviewed or are preliminary data for future publications, abstracts, posters, reviews, or meta-analyzes.

**Figure 2:**
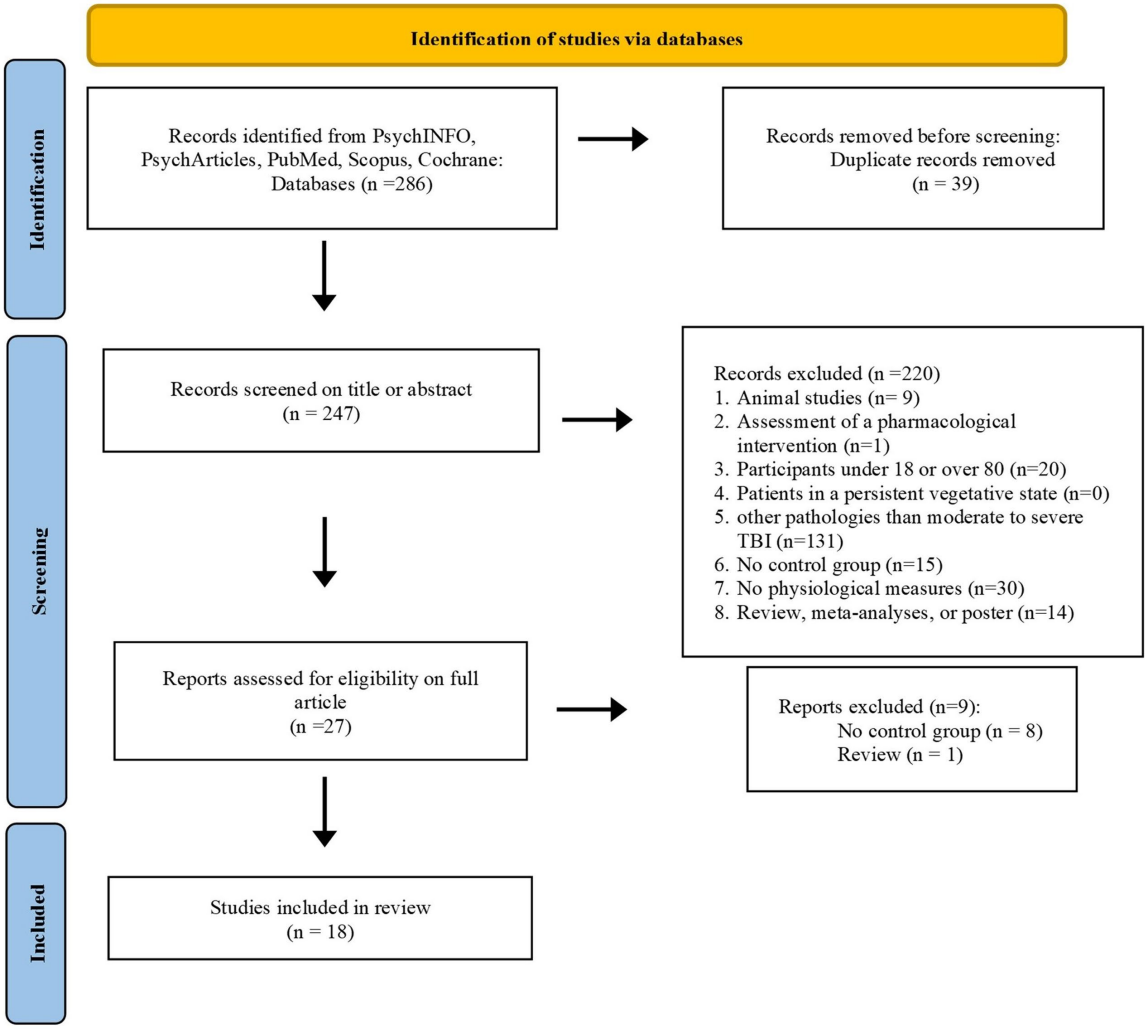
Flow diagram showing inclusion/exclusion of studies identified during database search process.

The research returned 286 articles from the six databases. We excluded 39 duplicates and screened 247 articles. Figure 2 summarizes the selection process and details of the reasons for articles’ exclusion. The first screening stage for titles and abstracts excluded 220 articles. The main reason for exclusion was the fact that the articles focused on mild TBI. A second screening stage on the full article excluded nine articles because of the absence of a control group. Eighteen articles were included in this review. Study selection was performed with two reviewers independently and was cross-checked by the two reviewers, and all disagreements in the team were unanimously resolved. For data extraction, a standardized data collection form was used by two independent researchers.

### 2.3. Quality assessment

To our knowledge, no evaluation criteria exist for non-randomized, non-interventional, and psychophysiological studies. We used Farrington’s suggestions for the assessment of methodological quality standards (Farrington, 2003) to develop our own criteria for the quality of the studies included in our review [see also the systematic review on PR in autism by Lydon et al., 2016]. We established 12 criteria (see Table 1), all of which were fulfilled by the 18 selected studies.

**Table 1:** Quality criteria.

Descriptive validity:	1. The design of the study is stated.
	2. The sample size is stated.
	3. Participant characteristics concerning age, gender, TBI severity and time post-injury are outlined.
	4. The physiological response, and any behavioral responses being measured, are operationally defined.
	5. The stimulus/stimuli are described in detail including information on their emotional content and duration.
	6. The study contained a physiological data baseline, and its duration is specified.
	7. If standardized measures are used, their psychometric properties are stated.
	8. Statistical methods employed are described.
Internal validity:	1. The study included a control group.
	2. Baseline physiological activity is considered during analyzes.
Statistical conclusion validity:	1. Statistical analyzes are appropriate for the research question and are performed with parametric tests.
	2. The statistical significance of the findings is stated.

## 3. Results

First, we present the sample characteristic, followed by physiological measures and stimuli. Second, studies’ results are presented according to the type of physiological measurement and stimuli used. Table 2 lists the characteristics of the participants, stimuli used, types of physiological measures, main results, and potential link with psychological assessment for each of the 18 studies.

**Table 2:** Summary of studies.

Author’s name	n	Age mean (± SD)	Severity of TBI	Time post injury mean (± SD)	Stimuli/task	Physiological measure(s)	Findings	Link with psychological assessment
Aboulafia-Brakha et al. (2016)	20 TBI, 22 controls	TBI: 37.4 (±12.6); Controls: 33.4 (± 8.5)	12 severe, 3, moderate, 5 mild	18.8 months (±12.01 months)	Verbal reports; Emotion faces on video clips	EDA; SCL	Anger regulation task: Only in the TBI group was mean SCL higher during uninstructed compared to neutral recall.	/
							Emotion recognition task: No difference between groups was observed	
Amorapanth et al. (2016)	16 TBI, 10 controls	TBI: 51.9(±15.0); Controls: 38.0 (± 14.4)	/	5.9 years (± 8.6 years)	Emotional film clips	ECG; HRV	TBI group exhibited lower LFA HRV during amusement films compared to controls. Conversely, the TBI group showed higher LFA HRV during sad films than the control group.	This increase in sympathetic activity for sad films was correlated with self-reported attentional difficulties and impairment in visual attention.
de Sousa et al. (2012)	21 TBI, 25 controls	TBI: 41.2 (±13.1); Controls: 29.0 (±11.1)	Severe	14.3 years (±10.4 years)	Emotional film clips	EMG; ZM and CM; EDA: SCL	ZM: Greater activity for pleasant films than neutral or unpleasant in controls, little differentiation between the type of films for TBI. Activity increased over time in the control group, especially for pleasant films, this activation decreased in the TBI group, especially for unpleasant films. CM: Greater activation for unpleasant films compared to pleasant and neutral films in controls, no effect valence in TBI. SCL: Lower SCL during unpleasant films in TBI compared to controls. Habituation during unpleasant films and sensitisation during pleasant films only in controls.	
de Sousa et al. (2010)	20 TBI, 22 controls	TBI: 47.4 (±10.0); Controls: 36.1 (± 12.6)	Severe	13.4 years (± 6.9 years)	IAPS pictures	EMG; ZM and CM; EDA: SCR	ZM: No difference between groups. CM: TBI showed reduced responses to unpleasant pictures. SCR: TBI group exhibited reduced SCR in pleasant, unpleasant, and neutral pictures compared to control participants.	ZM: No significant correlations with emotional or cognitive empathy for both groups. CM: Positive correlations with emotional and cognitive empathy scores for controls during unpleasant pictures. SCR: Positive correlation with cognitive empathy and with emotional empathy (trend toward significance) for TBI during pleasant pictures
de Sousa et al. (2011)	21 TBI, 22 controls	TBI: 48.4 (±8.8) Controls: 36.1 (± 12.6)	Severe	11.9 years (± 7.8 years)	Facial expressions pictures	EMG: ZM and CM; EDA: SCR and SCL	SCL: Reduced SCL to anger faces in TBI. SCR: Greater SCR to angry faces compared to happy faces in controls, larger SCR to happy faces.in TBI. ZM: No difference between groups. CM: produced greater activity for angry faces compared to happy faces in controls, with no emotional effect in TBI.	SCR: Same SCR to angry and happy faces for TBI and controls with higher emotional empathy level, participants with a lower level of empathy presented higher SCR for happy faces compared to angry faces. ZM: Larger ZM for happy faces than angry for the higher emotional empathy TBI and controls groups. No emotion effect for the low empathy TBI and control group. CM: Group effect with higher CM for TBI group comparing their control counterparts. Emotion effect and group effect in low empathy group: greater CM for angry faces compared to happy faces, inverse pattern in TBI group
Fisher et al. (2015)	19 TBI, 19 controls	TBI:44.89 (±13.76); Controls: 43.95 (±15.15)	Severe	12.37 years (±7.99 years)	Facial expressions pictures	EDA: SCL	Lower SCL in TBI not emotional effect	
Francis et al. (2016)	30 TBI, 30 controls	TBI: 45,73 (±13,68) Controls: 46,9 (±12,91)	Severe	14.03 years (± 8.74 years)	Resting state and one session of HRV biofeedback	ECG: HRV	No difference between groups at rest and during the biofeedback session	Positive correlation between HRV and social cognition and emotional empathy, negatively correlation with alexithymia in the TBI group.
Kelly et al. (2017)	21 TBI, 17 controls	TBI: 49.81 (±11.81); Controls: 46.29 (±16.22)	Moderate severe	at least 24 months post-injury.	Cyberball game	EDA: SCL	No difference	No correlation between SCLs and self-reported emotional experience in both groups
Krpan et al. (2011)	18 TBI, 24 controls	TBI: 38.8 (±13.6); Controls: 38.7 (± 17.4)	Moderate–severe	153 months (±117 months)	Simulated real-world stress test	EDA: SCL, ECG: HR, cortisol	SCL: No significant difference between groups. HR: No significant difference between groups Cortisol: No significant difference between groups	/
McDonald et al. (2011)	18 TBI, 18 controls	TBI: 49.0 (range 31 to63 years); Controls: 35.9 (range 20 to59 years)	Moderate severe	mean time since injury 13.0 (± 7.0 years)	Emotional faces pictures, passive and identification task	EDA: SCR and SCL, ECG: ECD	SCR: No difference between groups SCL: Passive task: increasing of SCL over trials for angry faces and decreasing for happy faces in controls Habituation and no emotion effect in TBI. Identification task: no habituation for either expression for either the control or TBI participants. ECD: Increasing for the identification condition compared to the passive condition in both groups. Identification task: magnitude increasing across repetition higher in controls relative to TBI. Increasing for the happy face across the repetition and decreasing for the angry face in controls, no emotional effect on TBI	No correlation between physiological measures and accuracy in recognizing tasks in both groups
Osborne-Crowley et al. (2020)	30 TBI, 30 controls	TBI: 44.47 (±15.32); Controls: 41.70 (±14.97)	Moderate Severe	13.63 years (±13.08 years)	Verbal report; Listening stories	EDA: SCL	Greater SCL across the emotional conditions in controls compared to TBI.	
(Rushby et al., 2013a)	17 TBI, 22 controls	TBI: 46.47 (±13.30); Controls: 41.23 (± 14.86)	Severe	12.59 years (± 8.05 years)	Resting state eyes open, eyes closed	EDA: SCL	Lower SCLs across both conditions in TBI compared to controls	
Rushby et al. (2016)	24 TBI, 24 controls	TBI: 43.3 years (± 14.96); Controls: 42.4 (±14.9)	Severe	12.63 years (±8.81 years)	Resting state	EDA: SCL	Lower SCL for the TBI compared to controls	
Rushby et al. (2013b)	19 TBI, 25 controls	TBI: 41.5 (±13.8); Controls: 31.0 (±11.1)	Severe	13.21 years (±10.3 years)	Emotional film clips	EMG: ZM and CM; EDA: SCL, ECG: HR	SCL: Increasing across films repetition in both groups. No emotion effect ZM: No difference between groups CM: Greater activity across all film types in TBI compared to controls. Emotion effect with higher activation for unpleasant films compared to neutral and pleasant films in both groups HR: Increasing across films repetition in controls, deceleration in TBI	SCL: Positive correlation with emotional empathy only in TBI ZM: Larger response in TBI for the low emotional empathy group compared to the higher empathy TBI group.
Sánchez-Navarro et al. (2005)	19 ABI* (14 TBI, 5 ischemic stroke), 23 controls	ABI: 31.63 (±13.05); control: 20.78 (±2.45)	Frontal damage	24.17 years (±2,01 years)	IPAS pictures	EDA: SCL and SCR; Startle blink, ECG: HR	SCL: No significant difference between groups SCR: Lower responses in TBI than in controls Startle blink: Lower responses in TBI for unpleasant pictures and higher ones to pleasant pictures compared to controls HR: No difference between group	
Saunders et al. (2006)	13 TBI, 24 controls	Adults	Severe	Mean: 6 years 9 months (range 1–17 years).	IAPS pictures, emotional faces pictures	Startle blink	IAPS pictures: Attenuation for pleasant pictures and potentiation for unpleasant ones in controls, attenuation for pleasant and unpleasant pictures in TBI. Slower to reach peak eyeblink response for positive and negative pictures in TBI compared to controls Faces pictures: no emotion and group effects	No correlation between subjective valence ratings and eyeblink peak, latency, or subjective arousal ratings in both groups
Soussignan et al. (2005)	1 TBI, 10 controls	TBI: 30, Controls: 26.4 (±4.87)	Severe	11 years	IAPS pictures	EMG: ZM and CM; ECG: HR; EDA:SCR	Pictures: SCR: Lower responses for all pictures in TBI. No emotion effect in TBI contrary to controls.ZM and CM: no emotional effect only in TBI Odors: SCL: No valence effect in TBI, greater SCL for unpleasant odors than pleasant or neutral ones in the control group. HR: No group and valence effect ZM and CM: no valence effect on TBI.	
Williams and Wood (2012)	64 TBI, 64 controls	TBI: 32.05 (±12.60); Controls 34.73 (± 12.14)	Moderate to severe	2.52 years (± 1.422 years)	IAPS pictures acoustic stimulus	Startle blink	Reduce effect of valence in TBI compared to controls	No correlation between with performance on europsychological measures in TBI

ABI, Acquired brain injury.

### 3.1. Sample characteristics

The sample size ranged from 11 to 128 participants, with a total of 386 patients with TBI, 5 patients with ischaemic stroke, and 431 control participants. The mean age of patients was 42.58 and 36.62 for control participants. We analyzed the pooled effect of age using the mean and standard deviation of both groups for 16 studies. Two studies were not included in this analysis because the authors did not specify the mean age and standard deviation (Saunders et al., 2006; McDonald et al., 2011). The pooled effect was medium (d = −0.34, SE = 0.0752, 95% CI 0.4841–0.189) and the heterogeneity between studies moderate (Cochran’s Q, df = 15, *p* = 0.0004, *I*^2^ = 40.2%; Hedges and Olkin, 1985). The patient sample included 290 men (74.17%) and 101 women (25.83%), and the control sample included 285 men (6.13%) and 146 women (33.87%). The percentage of women in the TBI sample was significantly lower than in the control group (M_TBI_ = 23.77%, SD = 12.59, M_controls_: 32.34%, SD = 15.66; W = 92, *p* = 0.028). Conversely, the percentage of men was significantly higher in TBI sample (M_TBI_ = 78.22%, SD = 15.47, M_controls_ = 67.66%, SD = 15.66; W = 88, *p* = 0.019).

Concerning the injury severity, 15 studies included only moderate to severe TBI (Soussignan et al., 2005; Saunders et al., 2006; de Sousa et al., 2010, 2011, 2012; Krpan et al., 2011; McDonald et al., 2011; Williams and Wood, 2012; Rushby et al., 2013a, 2013b, 2016; Fisher et al., 2015; Francis et al., 2016; Kelly et al., 2017; Osborne-Crowley et al., 2020), one study included mild to severe TBI (Aboulafia-Brakha et al., 2016). Sánchez-Navarro et al. (2005) included patients with frontal brain damage, and Amorapanth et al. (2016) included patients with TBI with cognitive or emotional disorders, but these two studies did not specify the severity of brain injuries. Finally, the mean time post-injury was 10.42 years, the shortest post-injury period was 3 months (Aboulafia-Brakha et al., 2016) and the longest was 40 years (de Sousa et al., 2012). Kelly et al. (2017) did not specify the average post-injury time and only mentioned a minimum time of 24 months post-injury.

### 3.2. Physiological measures

Table 3 summaries the physiological measures contained in each study. Most studies used several physiological measures (82.35%). The most commonly used measure was EDA in 14 studies (Soussignan et al., 2005; de Sousa et al., 2010, 2011, 2012; Krpan et al., 2011; McDonald et al., 2011; Rushby et al., 2013a, 2013b, 2016; Fisher et al., 2015; Aboulafia-Brakha et al., 2016; Kelly et al., 2017; Osborne-Crowley et al., 2020), followed by cardiac activity in 7 studies (Sánchez-Navarro et al., 2005; Soussignan et al., 2005; Krpan et al., 2011; McDonald et al., 2011; Rushby et al., 2013b; Amorapanth et al., 2016; Francis et al., 2016), and facial EMG in 8 studies (Sánchez-Navarro et al., 2005; Soussignan et al., 2005; Saunders et al., 2006; de Sousa et al., 2010, 2011, 2012; Williams and Wood, 2012; Rushby et al., 2013b). Krpan et al. (2011) measured cortisol levels, and Amorapanth et al. (2016) recorded the respiratory rate. As this respiratory measurement is used in combination with cardiac data to calculate the respiratory sinus arrhythmia (RSA), the last study will be discussed in Section 3.

**Table 3:** Summary of physiological measurements by study.

Author’s name	Table 1			EDA		EMG			Cortisol
	HR	HRV	RSA	SCL	SCR	ZM	CM	Blink reflex	
Aboulafia-Brakha et al. (2016)				X					
Amorapanth et al. (2016)		X	X						
de Sousa et al. (2012)				X		X	X		
de Sousa et al. (2010)					X	X	X		
de Sousa et al. (2011)				X	X	X	X		
Fisher et al. (2015)				X					
Francis et al. (2016)		X							
Kelly et al. (2017)				X					
Krpan et al. (2011)	X			X					**X**
McDonald et al. (2011)	X			X	X				
Osborne-Crowley et al. (2020)				X					
Rushby et al. (2013a)				X					
Rushby et al. (2016)				X					
Rushby et al. (2013b)	X			X		X	X		
Sánchez-Navarro et al. (2005)	X			X	X			X	
Saunders et al. (2006)								X	

### 3.3. Stimuli

The types of stimuli used to elicit PR vary between studies. Eight studies used emotional pictures (Sánchez-Navarro et al., 2005; Soussignan et al., 2005; Saunders et al., 2006; de Sousa et al., 2010, 2011; McDonald et al., 2011; Williams and Wood, 2012; Fisher et al., 2015), three of which presented pictures of emotional faces (de Sousa et al., 2011; McDonald et al., 2011; Fisher et al., 2015), four others presented emotional pictures (Sánchez-Navarro et al., 2005; Soussignan et al., 2005; de Sousa et al., 2010; Williams and Wood, 2012) from the standardized International Affective Picture System (IAPS, Lang et al., 1997), and the last one presented both types of pictures (Saunders et al., 2006). One study used short video clips of emotional faces (Aboulafia-Brakha et al., 2016). Three studies were extracted from famous movies (de Sousa et al., 2012; Rushby et al., 2013b; Amorapanth et al., 2016), two asked participants to report an event (Aboulafia-Brakha et al., 2016; Osborne-Crowley et al., 2020), two simulated social stress situations (Krpan et al., 2011; Kelly et al., 2017), three contained acoustic startle probes (Sánchez-Navarro et al., 2005; Saunders et al., 2006; Williams and Wood, 2012), and one used odor (Soussignan et al., 2005). Five studies included different types of stimuli (Sánchez-Navarro et al., 2005; Soussignan et al., 2005; Saunders et al., 2006; Williams and Wood, 2012; Aboulafia-Brakha et al., 2016), whereas the remaining studies included only one type of stimulus (de Sousa et al., 2010, 2011, 2012; Krpan et al., 2011; McDonald et al., 2011; Rushby et al., 2013a, 2013b, 2016; Fisher et al., 2015; Amorapanth et al., 2016; Francis et al., 2016; Kelly et al., 2017; Osborne-Crowley et al., 2020). Finally, three studies used measures at rest as the main measure (Rushby et al., 2013a, 2016; Francis et al., 2016) and seven others reported physiological data during the baseline (Sánchez-Navarro et al., 2005; Krpan et al., 2011; McDonald et al., 2011; Rushby et al., 2013b; Fisher et al., 2015; Aboulafia-Brakha et al., 2016; Kelly et al., 2017). In the following sections, studies are presented according to the type of physiological measurement and stimuli used.

### 3.4. Electrodermal activity

Two different indices were used to assess EDA in the reviewed studies: first, the skin conductance response (SCR) refers to a phasic change in the electrical conductivity of the skin in response to a change in an environment, such as emotional stimuli presentation. SCR is measured by the maximum amplitude change of the signal occurring in seconds following stimulation (usually between 1 and 3 s; Grapperon et al., 2012). SCR is used, for example, to measure habituation to repeated stimulus presentations. Second, the skin conductance level (SCL) refers to the tonic level of the electrical conductivity of the skin. This reflects general changes in autonomic arousal. It is characterized by slow and long-lasting state changes related to the accumulation or resorption of sweat in the surface layers. SCL increases (sensitisation) after stimuli presentation but rapidly decreases (habituation) when participants are attending passively. However, when participants are actively engaged in a task, habituation does not occur (Barry, 2004; Nagai et al., 2004). Among the 14 studies measuring EDA, 11 studies reported statistically significant differences between the responses of TBI and control participants (Sánchez-Navarro et al., 2005; Soussignan et al., 2005; de Sousa et al., 2010, 2011, 2012; McDonald et al., 2011; Rushby et al., 2013a, 2016; Fisher et al., 2015; Aboulafia-Brakha et al., 2016; Osborne-Crowley et al., 2020). For 9 of them, TBI participants showed a reduction in EDA compared to controls. Conversely, in the study by Aboulafia-Brakha et al. (2016), patients with TBI showed higher SCL during uninstructed anger recall than the control group. Finally, Kelly et al. (2017), Krpan et al. (2011), and Rushby et al. (2013b) did not report significant differences between TBI and control groups.

#### 3.4.1. Pictures

Seven studies measured EDA during image presentation; three used stimuli from the IAPS (Sánchez-Navarro et al., 2005; Soussignan et al., 2005; de Sousa et al., 2010), and the other used pictures or short video clips of facial expressions (Sánchez-Navarro et al., 2005; de Sousa et al., 2011; McDonald et al., 2011; Aboulafia-Brakha et al., 2016).

##### 3.4.1.1. International affective picture system pictures

In three IAPS studies, TBI participants displayed reduced SCR across pleasant, unpleasant, and neutral pictures compared to control participants (Sánchez-Navarro et al., 2005; Soussignan et al., 2005; de Sousa et al., 2010). However, the observed power of the group effect on SCR is only mentioned by de Sousa et al. (2010): the partial eta squared (η^2^p) was 0.35, which corresponds to a small effect (Cohen, 1988). Concerning a possible valence effect in patients with TBI, no differences were observed between pleasant, unpleasant, and neutral pictures in the studies by de Sousa et al. (2010) and Soussignan et al. (2005). However, Sánchez-Navarro et al. (2005) reported a valence × group effect: healthy participants produced greater SCR for pleasant and unpleasant pictures as compared to neutral pictures, while the TBI group showed larger SCR for pleasant pictures only, without any difference between unpleasant and neutral conditions. Moreover, de Sousa et al. (2010) found a significant positive correlation between SCR amplitude and cognitive empathy levels. Similarly, a marginally significant positive correlation was observed with affective empathy. Taken together, these results suggest that after TBI, image valence has less influence on SCR, which is instead predicted by empathy abilities.

Although these findings are interesting, variability also existed in the results of the control groups. Hence, compared to neutral images, control participants produced greater SCRs for positive images (de Sousa et al., 2010), negative images (Soussignan et al., 2005), or both (Sánchez-Navarro et al., 2005). However, these studies differed in at least three dimensions. First, stimuli were presented for 6 s in all three studies, but their number differed between the studies: de Sousa et al.’s (2010) study used 18 pictures (6 per category), while Sánchez-Navarro et al. (2005) and Soussignan et al.’s (2005) studies contained 54 (18 per category) and 90 pictures (30 per category), respectively. Second, the data transformation methods differed between the studies. Sánchez-Navarro et al. (2005) used a log transformation [log (SCR-1)] without a pre-stimulus baseline. Soussignan et al. (2005) subtracted the 2-s baseline pre-stimuli from the largest value averaged in the 2-s window after stimulation, and de Sousa et al. (2010) used a similar method but with a 1-s prepicture baseline. Finally, sample sizes differed, ranging from 10 in Soussignan et al.’s (2005) study to over 20 participants in the two other studies. Moreover, Sánchez-Navarro et al. (2005) included patients with frontal stroke in their TBI group. These methodological discrepancies may have led to inconsistencies in the results of the three control groups as well as in the TBI groups.

##### 3.4.1.2. Facial expressions

Four studies used facial expressions: de Sousa et al. (2011) and McDonald et al. (2011) included happy and angry faces; Fisher et al. (2015) added neutral faces; and Aboulafia-Brakha et al. (2016) used short clips in which actors portrayed anger, surprise, disgust, happiness, sadness, and fear or neutral faces. The type of task also varied between studies. In de Sousa et al.’s (2011) and Fisher et al.’s (2015) studies, participants passively viewed pictures. The task of McDonald et al. (2011) contained two conditions: first, participants passively viewed the pictures; then, they had to identify the emotion among the six other emotions. Aboulafia-Brakha et al. (2016) also proposed an emotional recognition task in which participants identified the emotion portrayed in a clip from a list of seven emotional labels. In the passive viewing condition, the TBI group of de Sousa et al. (2011) exhibited lower SCR for angry faces than the control group. Indeed, while the control group showed greater SCR to angry faces than happy faces, the opposite effect was observed in the TBI group with larger responses to happy faces. However, when emotional empathy was considered, this response pattern was only observed among participants with low emotional empathy. The TBI and control participants with normal emotional empathy levels presented the same SCR to angry and happy faces, suggesting that emotional empathy played a role in SCR to angry faces after TBI. However, during a passive viewing task, the TBI group of Fisher et al. (2015) presented a lower SCL for angry, happy, and neutral faces than the control group; however, no effect of the type of emotion was observed in either group. Conversely, Aboulafia-Brakha et al. (2016) reported an emotional effect on the SCL mean, but no difference between groups during active emotional recognition. In their passive task, McDonald et al. (2011) did not report differences involving group, condition, or emotion in SCR. However, the SCL trial mean exhibited a greater level for happy faces than for angry faces in the TBI group. Moreover, while control participants showed sensitisation (increasing SCL) for angry faces and habituation (decreasing SCL) for happy faces, TBI participants rapidly habituated to both emotions. Habituation effects disappeared in both groups during the attend condition, suggesting the influence of attention on emotional arousal. Taken together, these results suggest that increasing attentional demand allows the EDA to be normalized in TBI, and that the level of empathy may influence electrodermal reactivity. However, the methods of data transformation were not consistent between the studies. Indeed, de Sousa et al. (2011) and McDonald et al. (2011) first subtracted the 1,000 msec prepicture baseline from the 1,000 and 4,000 msec of the picture onset, and then used a log transformation [Log (SCR – skin conductance response +1)] to standardize the data. In contrast, Aboulafia-Brakha et al. (2016) and Fisher et al. (2015) did not use any data transformation methods.

#### 3.4.2. Films

de Sousa et al. (2012) and Rushby et al. (2013b) measured the EDA during pleasant, unpleasant, and neutral scenes from six movies. The data collection of these two studies occurred concurrently, and the first report focused on physiological responses to the first viewing of each film clip, while the second examined physiological patterns across five separate viewings of each film. de Sousa et al. (2012) analyzed the SCL data by dividing 90 s of film into 3-time intervals (0–30 s, 30–60 s, 60–90 s). For each interval, TBI participants exhibited less SCL during unpleasant films compared to control participants, while neutral clips produced similar SCL between groups. In addition, habituation to unpleasant films and sensitization to pleasant films were observed in the control group but not in the TBI group which had the same SCL across time. In Rushby et al. (2013b), the SCL increased across five film repetitions (sensitization) in both groups, but no significant effect of group or valence was observed. Moreover, in the TBI group, higher SCL correlated with higher self-reported emotional empathy. Separated analyzes according to emotional empathy levels in TBI groups revealed that increasing SCL across film repetition appeared only in TBI participants with normal to elevated self-report empathy scores, whereas TBI participants with low empathy scores maintained a low level of SCL across repetitions. Like de Sousa et al. (2011), the authors suggested a causal relationship between the loss of emotional contagion and loss of empathy for people after TBI. Finally, note that the data standardization methods used in these two studies were similar. In both studies, the 2 min resting baseline period was subtracted from the first 90 s time interval of each of the film clips presented. However, de Sousa et al. (2012) divided 90 s into three 30-s time intervals (0–30 s, 30–60 s, and 60–90 s). The SCL was calculated by subtracting the mean activity in the resting baseline period from the activity at each time interval.

#### 3.4.3. Emotion induction

Aboulafia-Brakha et al. (2016) and Osborne-Crowley et al. (2020) elicited emotions through the recall of emotional memories. Aboulafia-Brakha et al. (2016) investigated SCL during an anger regulation task. In this task, participants were asked to recall a self-experienced neutral and angering event aloud. In the latter, participants had to recall the same event in three different conditions, (1) without instruction, (2) while focusing on the emotional aspects and (3) while focusing on the perspective of the other involved person. The authors found that the mean SCL was higher during uninstructed anger recall than during neutral recall only in the TBI group. However, these results have not been replicated by Osborne-Crowley et al. (2020). In this study, participants first recounted emotional self-experienced events that evoked anger, happiness, and sadness. The second time, they heard three stories like their own and three stories based on the stories of other participants. Healthy participants exhibited greater SCL than TBI participants under all conditions. An effect of condition was observed in both groups with higher SCL for telling their story compared to listening to a similar or dissimilar story. However, no interaction between the group and emotion was observed. Note that Aboulafia-Brakha et al. (2016) included mild TBI whereas the Osborne-Crowley et al.’s (2020) sample contained only moderate to severe TBI. Contrary to de Sousa et al. (2010, 2011), who linked abnormal SCL for emotional expressions of anger and pleasant pictures after TBI with low self-reported empathy, Osborne-Crowley et al. (2020) found no difference between the self-reported emotional empathy of patients with TBI and controls. These authors suggest that after TBI, empathy is preserved despite reduced autonomic arousal. Finally, note that the data standardization of these two studies differed: Osborne-Crowley et al. (2020) took a baseline 10 s before the start of the recall which they subtracted from the first minute of the recall, while Aboulafia-Brakha et al. (2016) did not standardize the data with the baseline and used the SCL mean during 3 minutes of verbal reports.

#### 3.4.4. Social stress situation

Two studies used social situation paradigms to obtain more ecological data (Krpan et al., 2011; Kelly et al., 2017). Kelly et al. (2017) measured the SCL during a cyberball game. This internet-based social exclusion paradigm involves an inclusion condition in which the ball is equally shared with the participant and an ostracism condition in which the participant is ignored by the other (fictive) players and does not receive the ball tosses. The results showed different patterns of SCL between the TBI and control groups. Whereas the SCL increased during the ostracism condition in the control group, the SCL was higher during the inclusion condition in the TBI group. However, the difference was not statistically significant. However, the self-reported emotional experience of ostracism significantly differed between the groups; TBI participants felt less included in the inclusion condition than in the control group and similarly excluded in the exclusion condition. Given that these self-reported behavioral results were not correlated with SCL, these authors postulated a dissociation of self-report and physiological arousal after TBI. Krpan et al., 2011 measured SCL during a psychosocial stress test in which participants had to prepare and give a speech to an expert in communications and perform mental arithmetic while being video recorded. The video recordings during the speech preparation were then analyzed to compare the number of avoidance behaviors (e.g., reading magazines, playing puzzles, text messaging, staring into space) and planful behaviors (e.g. writing, reviewing the speech or writing, and appearing to think). The groups did not differ in SCL or self-reported stress levels during the psychosocial stress test. However, the TBI group exhibited more avoidant than planful behaviors, whereas the opposite was true for the control group. Therefore, contrary to Kelly et al. (2017), there was no dissociation between SCL and self-reported emotional experience, but the authors found a dissociation between behaviors and PR induced by stress. Finally, Kelly et al. (2017) reduced the SCL data with the difference between the mean value of the 2-min baseline period and the mean value for each 10-s epoch across the game; then, the data were standardized with a log transformation. However, Krpan et al. (2011) did not mention the reduction or standardization of the data.

#### 3.4.5. Startle acoustic

Only one study, by Sánchez-Navarro et al. (2005), measured SCR during an acoustic startle stimulus. The authors did not report any SCR difference between TBI and control participants during an acoustic stimulus of a 50-ms burst of white noise.

#### 3.4.6. Olfactory stimuli

In Soussignan et al.’s (2005) case study, participants smelt five pleasant, five unpleasant, and five neutral odors before rating the level of pleasure and intensity of the smell. Analyzes showed greater SCR for unpleasant odors than for pleasant and neutral odors only in the control group. Moreover, unpleasant odors produced higher SC changes in controls than in participants with TBI. However, pleasure and intensity ratings differed between participants with TBI and controls. Finally, SCR correlated with pleasure and intensity ratings in control participants, but SCR did not correlate with rating scores in patients with TBI. These results suggest a dissociation between PR and self-reported measures. Concerning the data standardization method, the authors subtracted the SCR at the 2 s SC level immediately preceding the stimulus onset from the largest value averaged in the 2-s window after stimulation.

#### 3.4.7. Resting state

Among the eight studies examining resting-state SCL (Sánchez-Navarro et al., 2005; Krpan et al., 2011; McDonald et al., 2011; Rushby et al., 2013b, 2016; Fisher et al., 2015; Aboulafia-Brakha et al., 2016; Kelly et al., 2017), half reported a lower level of SCL in the TBI group compared to the control group. Indeed, Rushby et al. (2013b) and Fisher et al. (2015) found a marginally significant lower SCL in their TBI groups during a 2 min resting condition. This difference was significant in studies by Rushby et al. (2016) and McDonald et al. (2011). The duration of Rushby et al.’s (2016) resting condition was 2 min. The resting data of McDonald et al. (2011) were derived in the 500 ms period immediately before stimulus onset. It is noteworthy that for this last study, baseline data were taken between two stimuli, while for the three other studies, data were taken at rest. Conversely, several studies showed no difference in SCL between controls and patients with TBI during baselines of 5 min (Krpan et al. (2011), 3 min (Sánchez-Navarro et al., 2005; Aboulafia-Brakha et al., 2016), or 2 min (Kelly et al., 2017).

### 3.5. Facial electromyography

The study of emotional reactivity by facial EMG is mainly based on the measurement of the contraction of the brow muscles, namely the corrugator supercilia (CR), the cheek muscles, namely the zygomaticus major (ZM), and the startle blink. Whereas the ZM contraction produces smiling expressions, the CR is the muscle above the eyebrows that brings them together and contracts during negative emotions such as grief or anger (Ekman and Friesen, 1978). The measure of the startle blink is based on a biphasic emotional theory, as negative stimuli activate the defence system, and positive stimuli activate the appetitive system (Lang et al., 1990). As blinking is an aversive reflex, it is potentiated for unpleasant stimuli and decreased for pleasant stimuli. Usually, an acoustic startle probe is presented together with the stimuli. It often consists of short bursts of white noise. Among the five studies measuring ZM and CM (Soussignan et al., 2005; de Sousa et al., 2010, 2011, 2012; Rushby et al., 2013b), only one reported a difference between the ZM responses of TBI and control participants (de Sousa et al., 2012). Conversely, regarding CR, only one study observed a similar response between groups (de Sousa et al., 2012). Finally, three studies investigating startle blinks found differences between groups (Sánchez-Navarro et al., 2005; Saunders et al., 2006; Williams and Wood, 2012).

#### 3.5.1. Pictures

##### 3.5.1.1. International affective picture system pictures

Among the five studies using IAPS pictures, de Sousa et al. (2010) and Soussignan et al. (2005) measured ZM and CR activity, whereas Sánchez-Navarro et al. (2005), Williams and Wood (2012), and Saunders et al. (2006) measured startle blinks.

###### 3.5.1.1.1. Corrugator supercilia and zygomaticus major activity

de Sousa et al. (2010) reported higher ZM activity for pleasant pictures than for unpleasant and neutral pictures in both groups. A main effect of valence was also observed for CR, with higher activity for unpleasant pictures than for pleasant and neutral pictures. Moreover, an interaction between valence and group emerged, with significantly lower responses to unpleasant versus neutral pictures and marginally reduced activity to unpleasant versus pleasant pictures in the TBI group compared to controls. Interestingly, TBI participants self-rated these unpleasant pictures as less unpleasant and arousing than controls. Finally, while a positive correlation was observed between emotional and cognitive empathy scores and CR to unpleasant pictures in the control group, no correlation was observed in the TBI group. According to the authors, impaired emotional responsivity is associated with the impairment of the empathy network. In a study by Soussignan et al. (2005), a valence effect was observed in the control group with higher CR activation for unpleasant pictures and higher ZM activation for pleasant pictures. In contrast, participants with TBI’s ZM and CM activity did not differ according to the valence of the picture. Notably, Soussignan et al. (2005) study examined only one patient with TBI, but it contained three times more pictures (54) than de Sousa et al.’s (2010) study. However, the data reduction methods used in these two studies were similar (see above).

###### 3.5.1.1.2. Startle blink

Sánchez-Navarro et al. (2005) found a main effect of valence on blink magnitude in both groups, with larger blinks for unpleasant pictures than pleasant ones. In the control group, the blinks were larger during unpleasant than neutral pictures and lower during pleasant than neutral pictures. In contrast, in the TBI group, the difference between the unpleasant and neutral pictures did not reach statistical significance, and no difference between the pleasant and neutral pictures was observed. Finally, the intergroup comparison revealed that TBI participants showed lower startle blinks to unpleasant pictures and higher responses to pleasant pictures compared with the control group. Williams and Wood (2012) also found a valence effect in the control group, with linear amplitude increasing for pleasant, neutral, and unpleasant pictures. This linear pattern was also observed in the TBI group, but the differences among the three conditions were not significant. Moreover, like Sánchez-Navarro et al.’s (2005) results, unpleasant pictures produced larger startle responses in the control group than in the TBI group; however, no group effect was observed for neutral pictures. However, unlike in the latter study, no group effect was observed for pleasant pictures. Finally, Saunders et al. (2006) showed a trend of increasing blink amplitude from pleasant to neutral and unpleasant pictures in the control group. However, the TBI group produced higher blinks for neutral pictures than for pleasant and unpleasant pictures. Owing to this unusual and unexplained result, the authors compared blinks to pleasant and unpleasant pictures in TBI and control participants. The control group displayed an attenuation of the startle eyeblink response for pleasant pictures and potentiation for unpleasant pictures, whereas TBI participants demonstrated an attenuated startle eyeblink for both pleasant and unpleasant pictures. Saunders et al. (2006) also measured eyeblink latency as an index of interest in pictures. The TBI group was significantly slower to reach peak eyeblink response for both positive and negative pictures than the control group. In conclusion, these three studies show that blink reflexes are less differentiated according to the valence of the pictures after TBI and are attenuated for unpleasant pictures. Note that Sánchez-Navarro et al.’s (2005) study contained five patients with stroke and used 54 pictures (18 per category), whereas Williams and Wood (2012) and Saunders et al. (2006) included only TBI patients and used only 15 pictures (5 per category) and 18 pictures (6 per category), respectively. Williams and Wood (2012) and Sánchez-Navarro et al. (2005) also present startle probes in the absence of pictures. Williams and Wood (2012) found no significant differences across groups during the baseline of 12 startle probes before the presentation of the pictures. Sánchez-Navarro et al. (2005) reported significantly larger startle blinks in the control group than in the TBI group; however, these measurements were taken during the interstimulus phases between two pictures. For data reduction, the three studies standardized the raw amplitude in *z* scores and transformed them to *t* scores.

##### 3.5.1.2. Facial expressions

Two studies measured EMG in response to the passive viewing of pictures of angry and happy faces (Saunders et al., 2006; de Sousa et al., 2011). First, de Sousa et al. (2011) measured the contraction of the ZM and CR muscles and observed that happy faces elicited greater ZM activity than angry faces in both groups. However, the control group exhibited greater CR activity for angry faces than for happy faces, whereas no emotional effect was observed in the TBI group. Analyzes according to the level of emotional empathy showed similar ZM responses for the higher empathy TBI group and the control group, with happy faces evoking larger ZM responses than angry faces. In contrast, no group or emotional effects emerged in the low-emotional-empathy group. Concerning CR activity, responses were higher for both faces in the normal emotional empathy TBI group than in their control counterparts. However, in the low emotional empathy group, whereas control participants exhibited greater CR activity in response to angry faces compared to happy faces, the TBI group demonstrated an inverse pattern, with higher CR activity in response to happy faces compared to angry faces. These authors concluded that the loss of emotional empathy after TBI could contribute to the lack of CR reactivity in response to angry faces. Second, Saunders et al. (2006) measured eyeblink startles and showed no group or emotional effects.

#### 3.5.2. Films

de Sousa et al. (2012) and Rushby et al. (2013b) studied facial reactivity during the same movie clips. As mentioned in the previous section, the data collection of these two studies occurred concurrently; the first paper focused on physiological responses to the first viewing of each film clip, while the second examined physiological patterns across five separate viewings of each film. de Sousa et al. (2012) reported a reduced facial response in the TBI group. Indeed, the control group exhibited greater ZM activity for pleasant films than for neutral or unpleasant films, whereas the TBI group showed no emotion effect. The same pattern was observed for CR: the control group displayed greater activation for unpleasant films than for pleasant and neutral films, while there was no valence effect in the TBI group. Interestingly, a different pattern in the two groups was observed for the mean change in ZM activation during the clips. While ZM activity increased over time in the control group, especially for pleasant films, it decreased in the TBI group, especially for unpleasant films. Unlike ZM, the same effect over time of increasing CR for unpleasant films and decreasing CR for pleasant films was observed in both groups. These authors suggest that the “contagion” effect was amplified with time, especially in the control group. Contrary to the results of de Sousa et al. (2012) and Rushby et al. (2013a) reported no changes in ZM activity in patients with TBI, as both groups exhibited greater ZM activity for pleasant clips than for neural and unpleasant clips. Contrary to expectations, separate analyzes according to the self-reported level of emotional empathy of TBI participants showed a larger ZM response for the group with low empathy scores than for those with normal to elevated self-reported empathy scores. Moreover, the TBI group exhibited greater CR activity across all film types than the control group. Finally, the valence effect, characterized by a higher CR activation for unpleasant films than for neutral and pleasant films, was observed in both groups. In their discussion, Rushby et al. (2013a) explained the contradiction of their results with those of de Sousa et al. (2012) by the fact that repeated watching might normalize the ZM reactivity in TBI participants. Moreover, repeated watching requires more attention after TBI, which is marked by a higher CR activity in this group. As mentioned in the EDA section, the data standardization methods were similar in both studies.

#### 3.5.3. Odors

In addition to the IAPS pictures task, Soussignan et al. (2005) measured facial reactivity during the olfactory task described above. Control participants exhibited a main effect of valence with higher CR activity for unpleasant odors and greater ZM activity for pleasant odors, but participants with TBI showed no difference between these conditions, consistent with their picture task results.

### 3.6. Cardiac measures

Across seven studies on cardiac measures, four measured HR (Sánchez-Navarro et al., 2005; Soussignan et al., 2005; Krpan et al., 2011; Rushby et al., 2013b), two measured HRV (Amorapanth et al., 2016; Francis et al., 2016) and one measured evoked cardiac deceleration (ECD; McDonald et al., 2011). HRV refers to the variation in the time interval between heartbeats. This is an interesting index of the influence of the ANS on HR (Laborde et al., 2017). The ANS comprises an activating sympathetic branch and inhibiting parasympathetic branch. These two branches are responsible for the acceleration and deceleration of the HR, respectively. HRV can be quantified using time-domain and frequency-domain methods. In time-domain methods, the standard deviation of interbeat intervals (SDNN) and the root-mean-square of interbeat intervals (rMSSD) are often used as a global measure of temporal variability. Frequency-domain methods disentangle the influence of the parasympathetic and sympathetic systems on HRV. The low frequency (LF) component of HRV reflects both parasympathetic and sympathetic influences while the high frequency (HF) component reflects the parasympathetic influence (Shaffer and Ginsberg, 2017). Respiratory sinus arrhythmia (RSA), which refers to the phenomenon of HR acceleration on inspiration and HR deceleration on expiration, is also an indication of the influence of the parasympathetic system on HRV. Finally, ECD reflects the orienting attentional reflex toward new stimuli. This is the difference between the pre-stimulus baseline period and the slowest HR obtained during the post-stimulus period of the epoch (Graham and Clifton, 1966). A small majority of the cardiac studies in this review found similar responses for participants with TBI and controls. Indeed, across the four HR and ECD studies, two reported statistical differences between groups (McDonald et al., 2011; Rushby et al., 2013b) while the rest found no significant differences (Sánchez-Navarro et al., 2005; Soussignan et al., 2005; Krpan et al., 2011). In the two HRV studies, significant differences were found in the frequency domain for LF and HF (Amorapanth et al., 2016). Another study reported high inter-individual differences in the TBI group, but no statistical difference between the groups (Francis et al., 2016).

#### 3.6.1. Pictures

##### 3.6.1.1. International affective picture system pictures

Two articles reported HR during the presentation of IAPS pictures. First, Sánchez-Navarro et al. (2005) reported no difference between groups, as unpleasant and pleasant pictures produced higher HR deceleration than neural ones in both groups. Moreover, no difference was observed between the HR deceleration elicited by pleasant and unpleasant pictures. However, in Soussignan et al.’s (2005) study, for both groups, only the unpleasant pictures produced higher deceleration than the pleasant and neutral ones. Like Sánchez-Navarro et al.’s (2005) findings, no statistical difference was observed between the TBI and control groups. As discussed, these studies both contained 54 pictures (18 per category), but de Sousa et al.’s (2010) data reduction method subtracted the baseline pre-stimuli, whereas Sánchez-Navarro et al. (2005) used a log transformation [log (SCR-1)]. Finally, as mentioned above, Sánchez-Navarro et al. (2005) included patients with frontal stroke.

##### 3.6.1.2. Facial expressions

McDonald et al. (2011) were interested in ECD that occurs after angry and happy face presentations in a passive viewing condition and an attended emotional recognition condition. In both groups, a conditioning effect was observed, with higher ECD in the attended condition than in the passive condition. The control and TBI participants exhibited ECD to the emotional faces, with no difference between angry and happy expressions in either group. However, in the attended condition, ECD magnitude increased across repetitions. This increase was greater in the control group than that in the TBI group. *Post hoc* analyzes revealed that the control group exhibited an ECD increase for happy faces across repetition and a decrease for angry faces, whereas no difference between faces over trials was observed in the TBI group. Finally, no correlation between ECD and emotion recognition accuracy was found in either the group or condition. The authors concluded that there was an improvement in the orientation reflex due to increased attentional demand after TBI. However, the relationship between this and the emotion recognition accuracy remains unclear.

#### 3.6.2. Movies

Amorapanth et al. (2016) measured HRV during clips that elicited amusement, sexual amusement, sadness, or fear, compared to neutral films. No difference between the groups was observed for parasympathetic activity during RFA. However, for sympathetic activity, participants with TBI exhibited lower LF during amusement films than control participants. Conversely, TBI participants showed a higher LF during sad films than the control group. This increase in sympathetic activity for the sad film was correlated with self-reported attentional difficulties and impairment in visual attention shifting during the rapid number naming test. The authors concluded that attentional difficulties may contribute to abnormal reactivity to sad stimuli. In Rushby et al.’s (2013b) study, participants viewed five repetitions of six 2-min film clip segments containing pleasant, unpleasant, and neutral content. In the control group, the average HR over time showed a small increase across film repetitions, whereas a large deceleration was observed in the TBI group. The authors explained this HR deceleration over time by the attentional effort involved in sustaining attention to repetitions. Taken together, these two studies seem to link cardiac reactivity and attentional difficulties following TBI. However, the comparability of patients in these two studies cannot be guaranteed. Indeed, the Rushby et al. (2013b) patient group contained only severe TBIs, but Amorapanth et al. (2016) did not specify the severity of the TBI and included patients with cognitive or emotional impairment. Moreover, the type and number of film clips used differed between the two studies. Indeed, both studies contained films from similar validated and normed sets; however, Rushby et al. (2013b) also included films used in previous research, not from validated sets. In addition, Amorapanth et al. (2016) used 11 different clips (two per emotional category and one neutral), whereas Rushby et al.’s (2013b) study only contained six clips (two per category). Finally, the data reduction methods used were different. Rushby et al. (2013b) computed the HR signals by subtracting the mean activity at baseline prior to that occurring in the first 90 s of each film. Amorapanth et al. (2016) first determined an interval of interest (IOI) which was the 30 s of the film clip that most strongly elicited the target emotion (the authors did not specify whether this IOI was determined based on self-reported data or physiological data). Second, RFA and LFA were standardized by dividing the baseline activity 30 s before IOI. Third, logarithmic transformation was performed for each variable.

#### 3.6.3. Odors

The study by Soussignan et al. (2005) did not show HR modulations in response to odors according to valence or group.

#### 3.6.4. Social stress situation

Krpan et al. (2011) measured HR during psychosocial test stress (see description above) and did not report differences between the groups. As expected, controls and participants with TBI exhibited increased HR when performing the psychosocial stress test compared with the 5 min’ baseline.

#### 3.6.5. Resting state

Three studies reported the cardiac data at rest. First, Francis et al. (2016) measured HRV at rest and during an HRV biofeedback session in which participants reduced their breathing to six breaths per minute. Concerning HRV at rest, the TBI group showed higher within-group variability in the temporal and frequency domains, especially for HF and LF/HF ratio. The authors presented this LF/HR ratio as an index of sympatho-vagal balance, but the accuracy of this measure has been questioned (Billman, 2013). After a log transformation value, no difference between the groups was observed in the time and frequency domains. In the TBI group, HRV at rest was correlated with self-reported measurements of alexithymia, empathy, emotion perception, and social cognition abilities. Specifically, SDNN, rMSSd, LF, and HF positively correlated with self-reported empathy and social cognition performance. SDNN and LF negatively correlated with self-reported alexithymia. The authors did not specify the values of these correlations for the control group. Finally, the HRV changes for the time and frequency domains during biofeedback were similar in both groups. Second, for HR data, Sánchez-Navarro et al. (2005) and Krpan et al. (2011) did not observe any difference between control and TBI during their 3- and 5-min baseline.

### 3.7. Cortisol

A single study, led by Krpan et al. (2011), measured cortisol levels and reported no difference between the groups before and after the psychosocial stress test (described above).

## 4. Discussion

This systematic review aimed to detail the main findings of 18 studies published between 2000 and 2021 and examine electrodermal, facial, cardiac, and cortisol reactivity to emotional stimuli, odors, social stressors, and at rest among individuals with moderate to severe TBI. The findings showed discrepancies depending on the type of physiological measures and stimuli. First, patients with TBI often showed reduced electrodermal responses compared to controls. Across the 14 studies measuring EDA, 11 studies reported lower EDA after TBI during picture presentations, movies, participants’ verbal report (Sánchez-Navarro et al., 2005; Soussignan et al., 2005; de Sousa et al., 2010, 2011, 2012; McDonald et al., 2011; Rushby et al., 2013a, 2016; Fisher et al., 2015; Aboulafia-Brakha et al., 2016; Osborne-Crowley et al., 2020), two studies using a social stress situation paradigm found no difference between groups (Krpan et al., 2011; Kelly et al., 2017) and one reported higher SCL in the TBI group during a verbal report task of anger event recall (Aboulafia-Brakha et al., 2016). Lower reactivity was also observed for CR contraction and blink reflex in EMG studies. Across five studies measuring CM, four reported lower CM activation in the TBI group during film or picture presentation (Soussignan et al., 2005; de Sousa et al., 2010, 2011, 2012). Concerning the startle blink, three studies showed that responses of patients with TBI were less differentiated according to picture valence and attenuated for unpleasant pictures (Sánchez-Navarro et al., 2005; Saunders et al., 2006; Williams and Wood, 2012). In contrast, patients with TBI displayed significantly reduced ZM activity in only one of four studies (Soussignan et al., 2005). Similarly, the impact of TBI on PR was not so clear in ECG studies; among the seven reviewed studies, only two reported lower HRV or ECD in the TBI groups (McDonald et al., 2011; Amorapanth et al., 2016), four found no difference (Sánchez-Navarro et al., 2005; Soussignan et al., 2005; Krpan et al., 2011; Francis et al., 2016) and one reported higher HR deceleration across film repetitions (Rushby et al., 2013b). Finally, the only study measuring cortisol levels did not show abnormalities in the TBI group (Krpan et al., 2011). To explain these PR inconsistencies, we will discuss the role of neurophysiological factors as well as emotional, sociodemographic, and methodological factors.

The defense system reduced activation in TBI patients is our first hypothesis, as they may experience reduced affective responsivity, particularly to aversive and unpleasant-negative stimuli (Saunders et al., 2006; de Sousa et al., 2010; McDonald et al., 2011; Williams and Wood, 2012). According to motivational theory, two dimensions of motivation compose emotional responses: defensive for aversive stimuli and appetitive for attractive stimuli (Lazarus, 1991). The physiological pattern of defensive reactions reflecting sympathetic activation to provide energy and facilitate adaptive behaviors such as attack or escape, involves limbic brain structures, including the amygdala (Adolphs, 2001). This last interpret signals from the environment as a threat that triggers an alarm signal, induced by specific ascending systems of the brainstem. First, through the hypothalamus, it activates the functions of sweat glands, resulting in EDA (Christopoulos et al., 2019). Although EDA is not exclusively related to the defence network, studies have reported that the direct stimulation of the amygdala generates an immediate increase in EDA (Lang et al., 1964; Inman et al., 2020). Second, facial motoneurons of the brain stem activate cranial nerve VII which produces contraction of the orbicularis muscle, resulting in a blink startle reflex (Kettle et al., 2006). Third, fibers descending from the brainstem innervate the preganglionic neurones of the sympathetic system. The activation of sympathetic fibers results from an increase in adrenaline in the bloodstream and causes the release of noradrenaline at sympathetic nerve endings. This produces measurable physiological responses, such as an increase in cardiorespiratory rate. Finally, the amygdala is involved in CR contraction. Indeed, electrical stimulation induces an increase in the EMG activity of the CR (Lanteaume et al., 2007). Moreover, increases in CR activity during negative picture viewing are associated with greater amygdala activity (Heller et al., 2014).

Although the TBI population is heterogeneous in terms of the location and severity of the injury, the frontal and temporal areas (including important limbic structures such as the amygdala) are particularly vulnerable to brain damage. The hypothesis of reduced affective responsivity in TBI could explain the lower CR activation and startle blinking together with the absence of ZM activity differences (Sánchez-Navarro et al., 2005; Saunders et al., 2006; de Sousa et al., 2010, 2011; Williams and Wood, 2012; Rushby et al., 2013b). CR and startle blinks are activated in response to unpleasant aversive stimuli. The ZM is activated in response to pleasant stimuli. Finally, the lower EDA observed in most TBI groups in the reviewed EDA studies is consistent with this aversive defence system disturbance.

The lack of difference between the groups in ECG studies could be explained by the implication of processes other than the aversive system in the cardiac reflex. Like EDA, CM activation, or the startle reflex, some cardiac reactions are implicated in the aversive defence system (i.e., the defence reflex). The defence reflex is characterized by heart acceleration to provide energy to facilitate adaptive behaviors such as attack or escape (Lang et al., 2000). However, other cardiac reflexes are also correlated with the attentional process, such as the orienting reflex. This orienting reflex causes heart deceleration. It is elicited by a novel stimulation to facilitate the attention and perception of the stimulus (Vila et al., 2007). Cardiac deceleration reflects the processing of new perceptual information. This type of reflex is less affected by the valence of the stimulus than is the defence reflex (Bradley, 2009). According to Phillips et al.’s (2003) neural model of emotional perception, attentional processes during emotional perception are mediated by the dorsal system. The identification of the emotional significance of stimuli and the production of the affective state are mediated by the ventral system which includes the amygdala. These reflexes depend on two different processes and the neural system. This dissociation between the orienting and defence reflexes could explain the discrepancy between the cardiac studies in our review. The orienting reflex was preserved after the TBI. This is consistent with the HR deceleration in both groups after the presentation of the pleasant and unpleasant pictures reported by Sánchez-Navarro et al. (2005). In McDonald et al. (2011), both groups demonstrated an increase in heart rate deceleration in the attend condition relative to the passive condition. However, in Soussignan et al.’s (2005) study, deceleration was not affected by the valence of emotion in the TBI group. Rushby et al. (2013b) observed HR acceleration and deceleration in the control and TBI groups, respectively.

However, given the unequal distribution between the study measures, these interpretations should be considered with caution. Indeed, across the 18 studies included in this review, 13 examined EDA measures, whereas only eight measured ECG or EMG and only one cortisol. It would be important to gather more data to identify a clear trend. Moreover, as a given emotional stimulus did not have the same effect on all physiological measures, future studies should take several measures simultaneously (Lydon et al., 2016). This refers to the directional fractionation phenomenon as described by Lacey (1967). Accordingly, a specific stimulation gives rise to a particular multidimensional activation pattern. For example, the orientation reflex results in an increase in EDA together with a deceleration of HR. In contrast, the increase in EDA is associated with an acceleration of HR during a defence reflex.

A second hypothesis would concern the level of empathy of the TBI participants involved in the studies. Empathy is crucial to understand and respond appropriately to the emotional experience of others (Decety and Ickes, 2011). Cognitive empathy refers to the ability to adopt another person’s point of view, while emotional empathy refers to the ability to experience affective reactions to the emotional displays of others. Emotional empathy implies a mimicry of the physiological reactions of the others and, accordingly, PR to emotional stimuli is an important component of emotional empathy (de Wied et al., 2006; Nummenmaa et al., 2008; Bogdanov et al., 2013; Sonnby-Borgström, 2002). The literature has shown a decrease of emotional empathy after a TBI (de Sousa et al., 2010, 2011; Williams and Wood, 2012) contributing to the behavioral disorders frequently reported in this population (Milders, 2019). Several studies in this review investigated the link between PR and empathy (de Sousa et al., 2010, 2011, 2012; Rushby et al., 2013b; Francis et al., 2016; Osborne-Crowley et al., 2020). However, the results differed between studies according to the group of participants. Indeed, de Sousa et al. (2011) and Rushby et al. (2013b) found a positive correlation between EDA and emotional empathy in TBI group only. In addition, de Sousa et al. (2011) revealed the same SCR pattern in the “normal” level of empathy group for both controls and TBI participants, while in the low level of empathy group, this pattern differed between control and TBI participant. These results suggested that a loss of empathy after TBI leads to abnormal PR. There would be two subgroups across the TBI population: one with a preserved level of empathy and PR and another with reduced empathy affecting their PR. The abnormal EMG responses found in the lower empathy TBI groups of Rushby et al. (2013b) and de Sousa et al. (2011) support this assumption that a loss of empathy after TBI impacts the PR. But some results contradicted the idea that TBI participants with higher emotional empathy have preserved PR like those of control groups. Indeed, the TBI participants of de Sousa et al. (2011) with “normal” levels of emotional empathy demonstrated higher CM contraction than controls. In addition, Osbourne did not find a difference in self-reported emotional empathy between TBI participants and controls, while the TBI participants exhibited reduced EDA. This intact emotional empathy in the TBI group highlights the heterogeneity of emotional profiles after a TBI. In their discussion, de Sousa et al. (2012) suggested that two profiles of emotional disorder can be found in the TBI population. One is characterized by a loss of emotional control and higher emotional empathy levels, the other by an impaired drive (or motivation) and lower levels of emotional empathy. The loss of emotional control can correspond to impulsive profiles while the loss of drive corresponds to apathetic profiles (Tate, 1999). Indeed, TBI can lead to various behavioral disorders that can range from general hypoactivity with apathy, abulia and loss of psychic self-activation to general hyperactivity with impulsivity, distractibility, and disinhibition (Godefroy, 2004). Apathy after TBI has been linked with lower PR (Andersson et al., 1999). Beyond the level of empathy, apathy and this involvement with PR should be more investigated. The decrease of empathy after a TBI could be the consequence of a general lack of interest and loss of motivation. The study of the relation between PR and apathetic profiles could contribute to understanding the discrepancies observed across the studies reviewed.

Related to this idea of the influence of apathy on PR after TBI, one explanatory factor for PR inconsistency across studies is the attentional demands of the tasks. Overall, the physiological differences between TBI and controls are more pronounced in studies involving passive tasks (Sánchez-Navarro et al., 2005; Soussignan et al., 2005; Saunders et al., 2006; de Sousa et al., 2010, 2011, 2012; McDonald et al., 2011; Williams and Wood, 2012; Rushby et al., 2013a, 2016; Fisher et al., 2015; Amorapanth et al., 2016). These differences tend to diminish for studies in which participants are active (Krpan et al., 2011; McDonald et al., 2011; Aboulafia-Brakha et al., 2016; Francis et al., 2016; Kelly et al., 2017). This trend suggests that attend tasks could overcome the hypo reactivity induced by apathy in TBI participants. Future research should consider the effect of attentional demand of the task on PR after TBI.

In parallel, the presence of psychopathological disorders in patients with TBI may also influence PR. For instance, brain lesions often result from a traumatic experience (fall, traffic accident, interpersonal violence, war injury) and post-traumatic stress disorder (PTSD) is particularly frequent in TBI patients (Williams et al., 2002). In the general population, PTSD affects 27% of patients with severe TBI (Bryant et al., 2000) but the frequency of PTSD may reach 89% in war veterans with mild TBI (Carlson et al., 2011). As this review is the first to focus on PR after TBI, we have chosen not to include studies focusing on PTSD. However, PTSD has been associated with hyperarousal (Weston, 2014) and future studies should consider PTSD symptoms and their impact on PR in TBI participants.

In addition, demographic factors, like age and gender, also have an impact on PR. Indeed, PR declines with age (Neiss et al., 2009) and women exhibited higher PR for emotional stimuli than men (Bianchin and Angrilli, 2012). In this review, we noticed differences in age and gender distribution across the TBI participants and control samples. First, TBI patients were generally older than control participants. The mean age was 43 years old for TBI and 37 for control participants. Since PR to emotional stimuli declines with age (Gavazzeni et al., 2008), future studies should further match their samples by age and control the influence of age on their PR results. Secondly, the number of men was higher in TBI samples than in the control samples. This difference is not surprising as TBI occurs more frequently in men. Indeed, TBI usually results from risk-taking behaviors (traffic accidents, contact and extreme sports) or accidents at work in male-dominated professions (i.e., construction, military occupations; Iverson et al., 2011; Colantonio, 2016). In Europe, the prevalence of TBI is significantly higher in men than in women (independently of age, severity, and mechanism of injury), ranging from 55% in Sweden in 2001 to 80% in Ireland in 2005–2007 (Peeters et al., 2015). In The United States, men are approximately 40% more likely to suffer a TBI than women (Coronado et al., 2012). But in our case, these gender-based differences could contribute to the lower PR observed in TBI participants, as men exhibited lower PR than women (Bianchin and Angrilli, 2012; Bari, 2020). Therefore, this lack of gender matching in the samples limits the interpretation of several studies. Accordingly, we recommend considering the effect of gender on emotional responses in future research. Specifically, the persistence of the gender effect on PR after moderate to severe TBI should be investigated.

The amount of time post-injury could also contribute to the PR discrepancies. Most of the studies in this review used an inclusion criterion of at least one-year post-trauma. However, five studies did not use this criterion and included patients less than one year after their injury (Sánchez-Navarro et al., 2005; de Sousa et al., 2011; Williams and Wood, 2012; Aboulafia-Brakha et al., 2016; Amorapanth et al., 2016). According to the neuroplasticity principles, the brain recovers and restructures itself after an injury (Nudo, 2013). After a TBI, spontaneous recovery, which refers to the recovery of neurotransmission in spared tissue near and remote from the site of injury (Levin, 2003), occurs within six months after the injury (Nakamura et al., 2009). However, training can also induce plastic changes in the brain occurring months to years after injury (Chen et al., 2010). Conversely, TBI induced irreversible neurodegenerative changes related to widespread brain atrophy. This atrophy progresses over several months and perhaps even years post-injury (Sidaros et al., 2009). Therefore, studies on PR should preferably include patients only one year after their injury to minimize the influence of these processes on PR.

The heterogeneity of patient injuries between samples may also explain PR discrepancies. While focusing on moderate to severe TBI, our review also integrates studies with patients with mild TBI (Aboulafia-Brakha et al., 2016) or ischemic stroke (Sánchez-Navarro et al., 2005), raising the question of the influence of these differences. Only Aboulafia-Brakha et al. (2016) report higher reactivity for TBI group compared to the control. Their inclusion of patients with mild TBI may contribute to this higher reactivity. Since neurocognitive sequelae and the dysfunction of the ANS highly vary regarding the severity of the TBI (Dikmen et al., 2009; Esterov and Greenwald, 2017; Hilz et al., 2017), each study should include only one type of TBI. Second, with a group including stroke patients, Sánchez-Navarro et al. (2005) reported the valence effect of IAPS pictures while this effect was attenuated in the related studies (Soussignan et al., 2005; Saunders et al., 2006; de Sousa et al., 2010; Williams and Wood, 2012), supporting the potential influence of stroke patients’ inclusion on this valence effect. Although with brain injuries of the same size and location, patients with TBI and stroke may experience equal neurological, cognitive, and psychological disorders (Castor and El Massioui, 2018), spontaneous recovery would be about three months shorter after a stroke than after a TBI (Chen et al., 2010). Therefore, it seems appropriate to balance samples between TBI and stroke patients if their injuries and deficit profiles are comparable and respect the one-year post-injury delay (see above). To ensure the recruitment of patients with the same profiles, we recommend assessing psychological and neurocognitive deficits and ensuring that the patients present lesions of the same size and location. This inclusion of studies that did not respect post-injury delay and homogeneity of patients group limited general inferences in our review, but it also permitted us to highlight the major role of both methodological issues in future research.

A last factor that could explain PR discrepancies is the divergence between data recording and recording methods across the studies. These divergences were particularly marked in EDA studies. Regarding data recording, we noted differences or lack of information about the sampling rate. The sampling rate refers to the number of samples of the signal taken per second and is measured in hertz (Hz). The data quality and the possibility of smoothing them depend strongly on the selected sampling rate, but several studies do not specify them. For those that did, most used a sampling rate of 100 Hz, while only Rushby et al. (2016) used 256 Hz. According to the Nyquist theorem, to accurately reproduce the signal the sampling rate must be at least twice as high as the highest frequency in the signal (Landau, 1967). The EDA is considered as a ‘slow measure’ with a maximal frequency of 35 Hz (Boucsein, 2012); therefore, a sampling of 70 Hz should be enough. However, if the analysis requires the separation of phasic waveforms from tonic signals, which is the case in most of the studies in this review, a sampling no lower than 100–200 Hz is recommended (Figner and Murphy, 2011). In addition, smoothing procedures are sometimes necessary to remove noise from the signal. These procedures, which involve down-sampling, have less impact on the quality of the signal at higher sampling rates. However, the studies in this review did not specify whether they used smoothing methods for EDA data. Given the low sampling rates used in these, these procedures could have an impact on the quality of their data. Therefore, we recommend using sampling rates of at least 100–200 hz. Furthermore, we encourage future studies to specify the values of the sampling rate used and, if applicable, the type of smoothing method used. Discrepancies were also noticeable in the data reduction methods. For example, for the three studies using IAPS pictures, Soussignan et al. (2005) and de Sousa et al. (2010) derived SCR from a pre-stimulus baseline, but its length differed by one second. Sánchez-Navarro et al. (2005) used log transformation [log (SCR-1)] without a pre-stimulus baseline. Regardless of the type of stimulus, this discrepancy in data reduction procedures appears in all EDA studies. Moreover, some authors did not use the baseline to reduce the data or did not specify it (Krpan et al., 2011; Fisher et al., 2015; Aboulafia-Brakha et al., 2016). This lack of methodological specification raises questions as it does not allow for the replicability of studies. Other studies used the baseline before the experiment (de Sousa et al., 2011, 2012; Rushby et al., 2013b; Kelly et al., 2017) or directly before each stimulus (Sánchez-Navarro et al., 2005; Soussignan et al., 2005; de Sousa et al., 2010, 2011; McDonald et al., 2011; Osborne-Crowley et al., 2020). Finally, some authors used log transformation in addition to baseline subtraction (Sánchez-Navarro et al., 2005; de Sousa et al., 2011; McDonald et al., 2011; Kelly et al., 2017). To the best of our knowledge, there is no recognized reference method for physiological data standardization. This is a matter of debate in the EDA literature (Caruelle et al., 2019). The guide issued by the Biopac MP36R & Acknowledge software (Braithwaite et al., 2013) provides some suggestions for EDA data standardization and normalization. First, it is important to distinguish between normalization and standardization methods. The foremost method is intended to correct the data for parametric statistical analysis. If the data are not normally distributed, it is recommended to apply logarithmic or square-root transformations for the SCL and SCR amplitude measurements. As SCR magnitude measurements include data of 0, the log SCR + 1 transformation is recommended for this type of measure. Second, standardization methods are corrections that reduce inter-individual variability and facilitate the comparison of data. There are two common standardization methods. The first is to subtract for SCL or divide for SCR the maximum data from the minimum data taken at rest or during a baseline. But this method is controversial for two reasons: (1) the minimum value depends on the sensitivity of the device and may not correspond to the true value, and (2) the maximum value is inconsistent, even within the same individual (Dawson et al., 2016). To avoid this problem, it is recommended to use a second method of transformation into standard values such as Z-scores, by taking the mean value and standard deviation (Ben-Shakhar, 1985).

To conclude, this review is the first systematic study of PR after moderate-to-severe TBI. This review highlights methodological discrepancies regarding the collection and analysis of physiological data and composition of participants with TBI. For each of these, we propose concrete proposals for improvement in future studies. Furthermore, this systematic analysis made possible to highlight the physiological divergence according to the type of measurement. Based on this, we hypothesized their link with brain injuries after TBI and their impact on the underlying emotional process. We also discuss the role of emotional, sociodemographic on PR. However, studies with multiple simultaneous physiological measures and more homogeneous and controlled TBI samples are needed to test these hypotheses. Finally, the study of PR is the first step in understanding emotional processes and underlying body–brain interactions. The assessment of interoceptive abilities, that is, the perception of the state of the body (Ceunen et al., 2016), is the next step. Indeed, in the emotional process, PR is only effective if the individual is aware of it (Damasio, 2000). Since interoception modulates emotional experience, future studies should also assess this dimension. This will allow for a better and more complete understanding of emotional disorders in this population.

## Data availability statement

The original contributions presented in the study are included in the article/supplementary material, further inquiries can be directed to the corresponding author.

## Author contributions

AB designed the study, searched the scientific literature, collected data, performed the analyzes, and drafted the manuscript. SI was an independent reviewer who conducted the cross-check. MR revised the manuscript. All authors have contributed to and approved the final manuscript.

## Funding

AB was funded by a doctoral award from the University of Mons. SI was funded by the National Heath Institute of Belgium (F.R.S.-FNRS, Rue d’Egmont 5–1000 Bruxelles, Belgium, grant number: 40008729).

## Conflict of interest

The authors declare that the research was conducted in the absence of any commercial or financial relationships that could be construed as potential conflicts of interest.

## Publisher’s note

All claims expressed in this article are solely those of the authors and do not necessarily represent those of their affiliated organizations, or those of the publisher, the editors and the reviewers. Any product that may be evaluated in this article, or claim that may be made by its manufacturer, is not guaranteed or endorsed by the publisher.
